# Investigating influential factors through crash frequency models considering excess zeros and heterogeneity: New insights into mountain freeway safety

**DOI:** 10.1371/journal.pone.0320135

**Published:** 2025-04-09

**Authors:** Liang Zhang, Zhongxiang Huang, Lei Zhu, Songtao Yang

**Affiliations:** School of Traffic and Transportation Engineering, Changsha University of Science and Technology, Changsha, China; Nanjing Forestry University, CHINA

## Abstract

The use of statistical modeling methods to quantify crash causation on mountain freeways is limited by crash data availability and technical challenges posed by excess zeros and heterogeneity, resulting in a lack of significant targeting of proactive crash prevention measures on mountain freeways. We collected multidimensional crash-related information on mountainous freeways in China, including road design characteristics, traffic conditions, pavement performance, and weather conditions. To overcome the challenges of excess zeros and heterogeneity on modeling techniques, we innovatively developed two new models: the Random Parameter Negative Binomial Lindley (RPNB-L) model and the Random Parameter Negative Binomial Generalized Exponential (RPNB-GE) model. The goodness-of-fit indicates that the RPNB-L and RPNB-GE models stand out among the six competing models, suggesting that the Lindley and GE distributions are conducive to portraying the multi-zero attributes, while the regression coefficients randomization treatment provides a deeper portrayal of heterogeneous effects. Moreover, the analysis reveals a considerable number of causes for crash frequency on mountainous freeways in China. These include several interesting results, such as special segments like tunnels and interchanges, Pavement Damage Condition Index (PCI) and the stormy rainfall (TR), which have not been extensively studied in previous research. The research results provided important reference values for the selection of active safety countermeasures for mountain freeways.

## Introduction

In China, road traffic crashes account for over 60,000 fatalities annually. Of particular concern are mountainous freeways characterized by complex terrain and variable weather patterns. Studies have revealed that per 100 kilometers, mountainous freeways demonstrate significantly higher safety risks compared to conventional freeways, with crash incidence rates, fatality rates, and injury rates measuring 3.0 times, 5.1 times, and 3.8 times greater, respectively [1]. In response to the critical issue of road safety, the Chinese government issued the Highway Project Safety Evaluation Specification (hereafter referred to as the Specification) in 2015. This document employs both qualitative and quantitative methods to systematically assess traffic safety conditions during various stages of freeway projects, including feasibility studies, preliminary design, construction drawing design, and project delivery and evaluation. In contrast to the comprehensive framework established in the U.S. Highway Safety Manual (HSM) [2], the current Specification, while identifying critical procedural elements for these stages, demonstrates three notable limitations: (1) absence of systematic methodological frameworks, (2) insufficient practical implementation mechanisms, and (3) critical technological gaps in road safety management. Particularly evident is the lack of advanced analytical capabilities for freeway crash investigations, including the application of crash prediction modeling techniques for probabilistic risk assessment and countermeasure evaluation. This paper aims to fill the gap by proposing a crash frequency modeling approach for Chinese mountainous freeways, which will provide a more complete and effective solution for the crash risk analysis and safety management of such freeways.

An appropriate crash frequency model is critical for road safety assessment and improvement. As a result of recent advances in data collection and modelling methods, a growing number of security factors are better understood and new security improvements are being implemented [1,3,4]. However, traffic crashes are complex events that involve the interaction of multiple risk factors, making understanding the causes of crashes sufficiently challenging. In particular, several challenges remain concerning the application of crash modeling methods to freeway scenarios in China: (1) The traffic safety analysis of Chinese freeways predominantly emphasizes geometric design features and traffic conditions. This focus is inconsistent with the reality that crash causes involve multi-dimensional factors related to driver, vehicle, road, and environment interactions. Such a narrow perspective inevitably undermines the reliability of safety evaluations [5,6]. (2) The absence of systematic characterization methods for key intrinsic attributes within crash databases-such as excess zeros and heterogeneity-raises concerns regarding the accuracy of crash frequency models and the reliability of analyses measuring safety effects [7,8]. (3) Limited by the deficiencies of modeling database and technology, the measurement and evaluation results of mountain freeways have gaps with the actual situation, and even completely contradictory conclusions have emerged [8,9].

**Fig 1:**
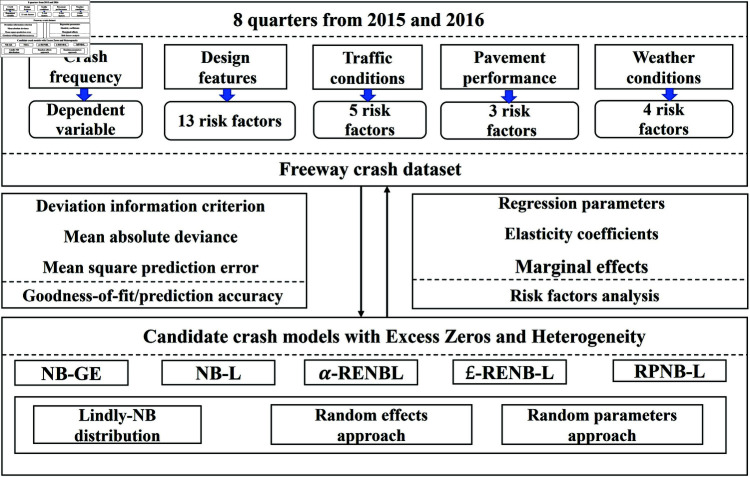
Pipeline of the proposed method.

Based on the questions raised above, this paper makes three key contributions (As shown in Fig 1): (1) A comprehensive crash frequency modeling dataset reflecting the real conditions of Chinese mountainous freeways has been developed, incorporating various risk factors such as road design characteristics, traffic volume and composition, pavement performance, and weather conditions. This dataset provides a standardized template for data collection in the safety management process of Chinese mountainous freeways. (2) A set of advanced crash frequency models has been developed by incorporating new distributions and parameter randomization techniques to address excess zeros and heterogeneity, thus expanding the library of freeway traffic safety analysis methods. (3) The safety factors relevant to Chinese mountainous freeways are identified from a fresh perspective, offering new insights into the analysis of crash causes. For instance, the safety effects of factors like climbing lanes and heavy rainfall warrant further investigation.

The structure of this study is as follows: The second section reviews the relevant literature. The third section details the database preparation and analysis process. The fourth section presents the proposed crash frequency models. The fifth section discusses the test results of these models and analyzes the causes of the crashes. Finally, the conclusion and future research directions are provided.

## Literature review

This paper summarized the latest research findings related to crash frequency modeling techniques and analysis of impact factors.

### Modeling technique of crash frequency

#### Modeling approach that deals with excess zeros.

The low probability events of accidents lead to an excessive number of zero-value samples (i.e. the dependent variable in the sample is zero) in the modelled dataset. This characteristic leads to a significant deviation between the actual accident frequency distribution and the traditional Poisson or negative binomial distribution, a discrepancy that is overlooked by conventional accident modeling methods. Studies [10,11] have shown that zero-inflated Poisson (ZIP) and zero-inflated negative binomial (ZINB) models, using a two-stage estimation process, offer substantial advantages in fitting data with multiple zero-value characteristics. However, these models have been criticized for focusing solely on maximizing statistical fit accuracy, while neglecting the underlying mechanisms of crash occurrence, which makes their application controversial [12].

Recently, new advancements in modeling approaches have been introduced to better handle excess zeros. These methods involve embedding distributions that are capable of handling small-count observations in traditional distributions such as Poisson or Negative Binomial distributions. This combination of distributions enhances the ability to handle zero-count observations, leading to improved model performance [13]. Prominent examples include Negative Binomial Lindley (NB-L) models [14–19] Negative Binomial Crack (NB-CR) models [20], Generalized Poisson Gaussian (PGG) Models [21] Poisson Weighted Exponential (PWE) Models [22], Poisson Inverse Gaussian (PIG) models [23,24], Negative Binomial Generalized Exponential (NB-GE) models [25,26] and Negative Binomial with Dirichlet Process [27]. Relevant studies also verified the advancement of such improved methods. For instance, Rusli et al. [15] compared the performance of the NB and the NB-L models using two crash databases with zero observation values of 89% and 90% respectively, which showed that the NB-L model had better goodness of fit and prediction accuracy.

#### Modeling approach that deals with heterogeneity.

The complexity of crash causes makes it impossible to account for all influencing factors, especially as the interactions between unobserved and observed factors vary across space and time-this phenomenon is known as heterogeneity [23,24]. Fixing the coefficients of all variables (e.g., Fixed Parameters models such as Poisson and Negative Binomial models) leads to biased estimates [24]. To address this limitation, several studies [25–30] introduced Random Effects (RE) models, embedding random terms into the link function to capture heterogeneity across samples. These studies demonstrated that RE models outperform Fixed Parameters models. However, such models cannot fully explain the complex impacts of unobserved heterogeneity by merely embedding a random term [30–32]. In view of this defect, the randomization of regression coefficients, known as Random Parameters (RP) models, was proposed to better characterize heterogeneity across samples [33–38]. The RP models do not regard the error term in the link function as the only random component, but allows the regression coefficient of each variable to change among samples, which more comprehensively describes the heterogeneity. The above research results also verified that the RP models have significant advantages over the traditional Fixed Parameters models and RE models in the field of road safety analysis, which provides a reliable reference for the selection of the crash frequency models of Chinese mountain freeways.

### Cause analysis of freeway crashes

#### Design features.

The road design features associated with crash frequency mainly include horizontal and vertical alignment indicators, cross-sectional indicators, speed limits and segment types. Several studies [30,31,39–41] have shown that the curvature, slope and latitude are positively correlated with the collision frequency, that is, the higher the above indicators, the higher the crash risk of the segments. However, there is some debate regarding the traffic safety effects of cross-sectional indicators. The studies [42–44] found that adding lanes would help reduce single-vehicle and multi-vehicle crashes, while Hou et al. [31] drew the opposite conclusion that three-lane and four-lane would lead to the probability of lane changing and overtaking compared with two-lane, thus increasing the risk of crashes. With respect to the safety effects of speed limits, freeways with higher speed limit values tend to have higher crash risks [43,45], and in particular, the speed limit difference between cars and trucks also had significant positive correlations with crash frequency [45]. Special segments of freeways, such as interchanges, tunnels, and service areas, have also been shown to have significantly higher crash frequency than those of ordinary freeway segments [14].

#### Traffic conditions.

Traffic condition indexes that influence traffic safety primarily include traffic flow, traffic composition, and traffic speed. The conclusion that traffic volume was significantly positively correlated with crash frequency has been confirmed by numerous studies [5,6,46–54]. Regarding traffic composition, Ding et al. [4] and Wen et al. [55] found that freeway segments with higher proportions of conventional cars and heavy vehicles tend to have less stable traffic systems, thereby increasing the crash risk. This is because the presence of heavy vehicles and conventional cars reduces the responsiveness of traffic flow, making it harder for drivers to adjust to environmental change. In addition, Yu et al. (2013) [53], Wen et al. [56] and Cai et al. [7] have shown that high average speeds of traffic flow on mountain freeways lead to a reduction in crash frequency, as drivers are more likely to react and respond to changing conditions when traveling at higher speeds. This is especially true during the dry season, when the effects of higher speeds on crash frequency are more pronounced.

#### Pavement performance.

In recent years, with the progress of detection methods, pavement performance indicators have been proved to have significant safety effects. Flask et al. [57] and Alnawmasi et al. [58] shown that vehicle subvert accidents are likely to occur when driving in segments with deep ruts. In addition, the interactions between the friction coefficient of pavement and rains significantly affected the traffic safety of freeways, that is, increasing the road friction coefficient significantly reduces the occurrence of crashes in rainy days [31]. The traffic safety effects of the International Flatness Index were controversial, Chen et al. [34] have shown that increased pavement flatness improves driving stability and reduces crash risks. In contrast, Behnood and Mannering [39] believed that uneven pavement instead forces to drive more carefully, thus contributing to improved traffic safety.

#### Weather conditions.

In recent years, the effects of meteorological conditions such as high winds, rainfall, temperature, and visibility on traffic safety on motorways have been thoroughly explored by numerous scholars [12,44–46,49]. The mainstream conclusions include: precipitation and visibility have significant negative impacts on traffic safety on freeways [44], especially heavy rainstorms greatly increase the probability of accidents [42]. Developed countries have further studied the traffic safety effects of micro-weather database or weather conditions at the time of crashes [12]. However, such studies have high requirements on the distance between meteorological observation stations and data collection systems, while road systems in general low- and middle-income countries hardly have the hardware and software conditions to meet the requirements.

### Summary of research and work direction

Research on freeway safety in developed countries, such as Europe and America, has been ongoing for many years and has yielded fruitful results. However, there have been few targeted studies on the specific driving culture and driving environment of Chinese mountain freeways. Based on this, the following problems should be addressed gradually.

- **The quality of the databases needs to be further improved.**
  Although European and American countries have specifically discussed the traffic safety effects of freeway design characteristics, traffic conditions, pavement performance, and weather conditions, little research has been conducted to incorporate all these potential risk factors into the traffic safety analysis of Chinese mountain freeways.
- **Excess zeros and heterogeneity should be given adequate attention.**
  Excess zeros and heterogeneity have been the main factors impacting the performance of crash models. The vast differences in freeway design concepts and management regulations between China and Europe and the United States make the validity of transplanting advanced crash models from Europe and the United States to Chinese mountainous freeways yet to be verified. Thus, it is an urgent necessity to provide reliable and improved modeling ideas for excess zeros and heterogeneity based on the actual conditions of mountain freeways in China.
- **The reliability of traffic safety analysis of Chinese mountain freeways needs to be improved.**
  At present, traffic safety analysis of Chinese mountain freeways is partly based on practitioners’ long-term working experience, and partly based on the results of qualitative safety evaluation methods of inference. Only a few are summarized based on the estimation results of a series of traffic safety econometric models. There is still scope for improvement in the traffic safety analysis methods for the mountainous freeways in China.

## Date preparation

### Research objects

We collected the crashes and potential risk factors of the Kaiping-Zhanjiang segments of the G15 freerway and the Lianzhou-Sanshui segments of the G55 freeway, which are located in the mountainous areas of southern China. The study objects are respectively undertaken by five management companies for daily operation, with a total length of 611km, and the roadway contains 50 interchanges, 8 service areas, 985 bridges and 50 tunnels (The basic conditions of each expressway are shown in Table 1).

**Table 1:** Summary information of the study subjects.

Freeways	Length (km)	Design speed (km/h)	Number of Interchanges	Number of service areas	Number of tunnels
Kaiyang	125	100	12	2	0
Yangmao	79	100	16	1	0
Maozhan	102	120	8	1	0
Lianhuai	186	100	14	2	20
Huaisan	116	120	8	3	30
Total	611	–	50	8	50

### Sample division methods

The quality of the crash modeling dataset was largely influenced by the temporal and spatial division of the samples [7]. Regarding the time scale, we chose to divide the data into ‘quarterly’ units for the following reasons: (1) potential risk factors such as traffic conditions, meteorological conditions, and pavement performance on mountainous freeways in China exhibit significant seasonal clustering and correlation [30], and (2) the quarterly division criterion prevented the generation of excessive zero-value samples compared to the micro-division criterion, represented by ‘month’, thus reducing some of the limitations of the crash model [32]. Based on the meteorological characteristics in southern China, February-April, May-July, August-October, and November-January of the following year were designated as the 1st-4th quarters of the year, respectively. This study collected crash and risk factor data over 8 quarters between 2015 and 2016.

**Fig 2:**
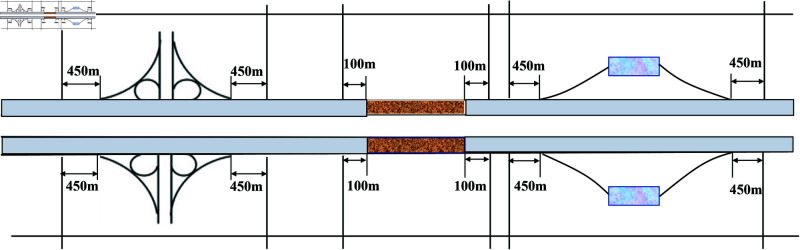
Segments type definition and division.

Referring to the clue provided by Hou et al. [6], the spatial division of the samples was performed using the uncertain segment length method. This approach focused on maintaining consistency of indicators within the same segment, while allowing for variability in segment length. It effectively addresses concerns about data authenticity that may arise from secondary processing of indicators. Specifically, we truncated the segments at locations where the segment type, plane geometry, slope, and number of lanes changed according to specific rules [33]: (1) Plane geometry indicators were classified into curved and straight sections, with smooth curves included in the curved category; (2) Locations with a longitudinal slope gradient change of  ≥ 1% were used as cut-off points; and (3) Segment types included basic segments, interchanges, service areas, and tunnel segments (the extent of each segment type is shown in Fig 2).

A total of 5568 (i.e. 696*8) samples were obtained by the above spatio-temporal division method.

### Description of variables

The modeling variables involved 26 risk factors, including traffic crashes, design characteristics, traffic conditions, pavement performance and weather conditions. With the above established samples as the carrier, the representation indicators of each variable were matched to the samples to form a complete modeling database. The description and statistical characteristics of the database are shown in Table 2.

The variables used for the model developments are extracted through the following task pipeline:

(1) The traffic crashes were obtained from the database maintained in each freeway management center, which detail the locations, causes, related vehicle information, severity of the crashes and casualties. A total of 4155 crashes were recorded.
(2) Road design indicators were mainly obtained through data collection and field investigation, including the construction design drawings and record documents provided by freeway management companies. Regarding the field research, the information of geometric features and traffic facilities were obtained by manual observation and validated with driving recorders. A total of 13 risk factors related to design indicators were recorded.
(3) China’s networked freeway toll collection system categorizes vehicles into five types based on their number of axles, wheels, wheelbase, and height (as shown in Table 3). In this study, the quarterly percentages of the three types of vehicles were excluded, considering their covariance with other variables. The formulas for calculating the quarterly average daily traffic (QADT) and the weighted proportion of each type of vehicle are shown in Eqs (1)–(5).
  $$\begin{array}{ccc} & {QADT}_{k,t}=\frac{V_{k,1,t}+1.5V_{k,2,t}+2V_{k,3,t}+{3V}_{k,4,t}+{3.5V}_{k,5,t}}{Q_{t}} & \end{array}$$ (1)
  $$\begin{array}{ccc} & {VP}_{k,1,t}=\frac{V_{k,1,t}}{V_{k,1,t}+1.5V_{k,2,t}+2V_{k,3,t}+{3V}_{k,4,t}+{3.5V}_{k,5,t}} & \end{array}$$ (2)
  $$\begin{array}{cc} & {VP}_{k,2,t}=\frac{V_{k,2,t}}{V_{k,1,t}+1.5V_{k,2,t}+2V_{k,3,t}+{3V}_{k,4,t}+{3.5V}_{k,5,t}} \end{array}$$ (3)
  $$\begin{array}{cc} & {VP}_{k,4,t}=\frac{V_{k,4,t}}{V_{k,1,t}+1.5V_{k,2,t}+2V_{k,3,t}+{3V}_{k,4,t}+{3.5V}_{k,5,t}} \end{array}$$ (4)
  $$\begin{array}{cc} & {VP}_{k,5,t}=\frac{V_{k,5,t}}{V_{k,1,t}+1.5V_{k,2,t}+2V_{k,3,t}+{3V}_{k,4,t}+{3.5V}_{k,5,t}} \end{array}$$ (5)
  where, $V_{k,1,t}-V_{k,5,t}$ represent the number of vehicles of category 1 to 5 passing through the toll section in quarter *t* (*t* = 1 , 2 , … 8). $Q_{t}$ represents the number of days in the quarter *t*. ${QADT}_{k,t}$ represents the average daily standard traffic flow of segment *k* in quarter *t*. ${VP}_{k,1,t}$, ${VP}_{k,2,t}$, ${VP}_{k,4,t}$ and ${VP}_{k,5,t}$ represent the proportions of class 1, 2, 4 and 5 vehicles in segment *k* and quarter *t*, respectively.
(4) The spatial and temporal collection frequencies of pavement performance indicators were 20/50m and 3–9 months, respectively. Therefore, the pavement performance metrics were matched to the samples by interpolation and averaging processes [7].
(5) According to the Standard for Evaluation of Highway Technical Condition (JTG H20-2007) [23], the pavement condition index (PCI) characterizes the degree of pavement damage, and a higher PCI represents a smaller degree of pavement damage. Ride Quality Index (RQI) characterizes the smoothness of the pavement, and the higher its value, the better the smoothness of the pavement. Skidding Resistance Index (SRI) is converted from the lateral force coefficient of the pavement, where the higher its value, the better the slip resistance of the pavement.
(6) Collected meteorological data, such as annual/monthly rainfall, average/maximum/ minimum temperatures, and other easily obtainable weather conditions data, are widely used in road traffic safety studies [7,55,56]. For example, the results of Wen et al. [56] showed that an increase in monthly average wind speed, monthly average daily precipitation, and monthly average visibility were detrimental to freeway traffic safety. Cai et al. [7] showed that the higher the ratio of light/moderate and heavy/stormy rainfall, the higher the crash frequency in freeway tunnels, with the most pronounced effect observed for heavy/stormy rainfall. Based on this, The weather condition variables selected in this paper include the percentage of small/moderate rain days in a quarter (SMR), the percentage of torrential rain days in a quarter (TR), the percentage of days with no sustained wind in a quarter (WD), and the per-centage of days with wind power  ≥  4 in a quarter (WP). The weather data used in this study were obtained from the Guangdong Meteorological Data Center and recorded at the county (district) level, and are covered by data from 41 meteorological stations, with an average distance of 36 km between stations. The shortest and longest distances between a meteorological station and a freeway are 18 km and 34 km, respectively.
(7) No collinear pairs used in modeling after the collinearity test.

**Table 2:** Summary information of the study subjects.

Abbr.	Description	Continuous Variables			Discrete variables	
		Mean	SD	Min/Max	Counts	Percentage(%)
**Dependent variable**						
NC	Number of crashes	0.746	1.383		–	–
**Freeway design features**						
SL	Segment length	0.877	0.31	0.172/2.691	–	–
ST	**Segment types**					
	0, Basic segments	–	–	–	4048	0.727
	1, interchange, tunnel or service segments.	–	–	–	1520	0.273
MBW	**Middle band width**					
	0, 1.5m	–	–	–	532	0.096
	1, 2m or 3m	–	–	–	5036	0.904
TER	**Truck escape ramp**					
	0, Do not include truck escape ramp	–	–	–	5550	0.997
	1, Including truck escape ramp	–	–	–	18	0.003
CL	**Climbing lane**					
	0, Do not include climbing lane	–	–	–	5428	0.975
	1, including climbing lane	–	–	–	140	0.025
PT	**Pavement types**					
	0, cement pavement	–	–	–	5392	0.968
	1, asphalt pavement	–	–	–	176	0.032
SLI	**Speed limit**					
	0, Non-speed-limiting segment	–	–	–	3927	0.705
	1, Speed -limiting segment	–	–	–	1641	0.295
DS	**Design speed**					
	0,100km/h	–	–	–	2040	0.366
	1,120km/h	–	–	–	3528	0.634
CUR	Curvature (1/km)	0.232	0.276	0/1	–	–
CUL	Curve length (km)	1.851	1.182	0.134/3.091	–	–
SLO	Slope (%)	0.866	2.073	-4/3.968	–	–
SLL	Slope length (km)	0.922	2.088	0/4.118	–	–
PRB	Proportion of bridge	0.32	7.603	0/1	–	–
**Traffic conditions**						
In QADT	Quarterly average daily traffic volume (veh/day)	9.421	1.21	8.786/9.849	–	–
IVE	Quarterly percentage of Class 1 vehicles	0.741	0.042	0.630/0.836	–	–
IIVE	Quarterly percentage of Class 2 vehicles	0.027	0.015	0.008/0.050	–	–
IVVE	Quarterly percentage of Class 4 vehicles	0.024	0.014	0.005/0.053	–	–
VVE	Quarterly percentage of Class 5 vehicles	0.12	0.047	0.051/0.227	–	–
**Pavement performance**						
PCI	Pavement condition index	96.798	4.312	62.442/100	–	–
RQI	Riding Quality Index	94.014	2.946	65.919/99.383	–	–
SRI	Skidding Resistance Index	91.801	4.534	63.75/99.1	–	–
**Weather conditions**						
SMR	Quarterly percentage of days with light/medium rainfall	0.421	0.149	0.135/0.663	–	–
TR	Quarterly percentage of days with Torrential rains	0.066	0.057	0/0.187	–	–
WD	Percentage of days with no sustained wind days in a quarter Quarterly percentage of days with no sustained wind	0.846	0.095	0.67/0.978	–	–
WP	Quarterly percentage of days with wind power ≥ 4	0.173	0.2	0.090/0.890	–	–

**Table 3:** Vehicle classification standard.

Vehicle types	classification standard				Typical vehicles
	wheel base	Height of head/m	number of axles	Number of tires	
5	≥ 3 . 2	≥ 1 . 3	> 3	> 10	40 foot container vehicles, Heavy trailers, Heavy trucks
4	≥ 3 . 2	≥ 1 . 3	3	6-10	20 foot container vehicles, Large trucks, Large trailers, Large luxury buses
3	≥ 3 . 2	≥ 1 . 3	2	6	Large ordinary buses, Medium trucks, Medium buses,
2	≥ 3 . 2	≥ 1 . 3	2	4	Minibus, Light trucks, Minivans,
1	< 3 . 2	< 1 . 3	2	2–4	Pickup trucks, Cars, Motorcycles,

## Methodology

### Innovation crash models considering excess zeros

**Fig 3:**
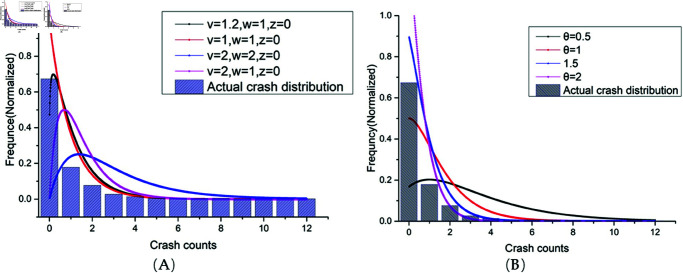
Resemblance of Lindley and GE probability distributions to the frequency distribution of observed crash counts with excess zeros, where *θ* represents the Lindley parameter and *ω*, *v* represent the scale parameter and shape parameter of GE distribution. (A) Resemblance of Lindley to crash frequency distribution. (B) Resemblance of GE to crash frequency distribution.

The necessity of introducing the new distribution was resolved by comparing the probability density profile distributions (shown in Fig 3). The Lindley and GE distributions have similar characteristics, i.e., the mean is close to 0 and the observations far from 0 have a long-tailed characteristic, which fits extremely well with the trend of the distribution of the actual crash frequencies. Therefore, the introduction of the two new distributions allows for an overall shift of the single NB distribution curve towards zero values, which is effective in capturing more zero-valued samples while retaining the advantage of the logic of the NB distribution in explaining crashes.

The core distribution of the NB-L model can be expressed as a hierarchy of NB distribution, Bernoulli distribution and Gamma distribution. Assuming that the crash frequency of segment *i* in time *t* follows the NB distribution, the probability distribution of $Y_{i,t}=y_{i,t}$ is:


$$\begin{array}{ccc} p\left(Y_{i,t}=y_{i,t}\right)={\left(\frac{1∕\alpha }{1∕\alpha +\eta_{i,t}}\right)}^{1∕\alpha }\frac{\Gamma (1∕\alpha +y_{i,t})}{\Gamma (1∕\alpha )y_{i,t}!}{\left(\frac{\eta_{i,t}}{1∕\alpha +\eta_{i,t}}\right)}^{y_{i,t}} & & \end{array}$$ (6)

where, *α* is the overdiscretized parameter of NB distribution; $\eta_{i,t}$ is the mixed distribution parameter of segment *i* in time *t*, which can be decomposed into the product of random parameter $\lambda_{i,t}$ and random parameter $\tau_{i,t}$:


$$\begin{array}{ccc} \eta_{i,t}={\tau_{i,t}\lambda }_{i,t} & & \end{array}$$ (7)

where, there is exponential correlation between $\lambda_{i,t}$ and crash risk factor vector $X_{i,t}$:


$$\begin{array}{ccc} \lambda_{i,t}=\exp(\beta X_{i,t}+\varepsilon_{i,t}) & & \end{array}$$ (8)

where, *β* is the regression coefficient vector corresponding to $X_{i,t}$. While $\tau_{i,t}$ follows Lindley distribution, and its probability density function is:


$$\begin{array}{ccc} f\left(\tau_{i,t},\theta_{i,t}\right)=\frac{{\theta_{i,t}}^{2}}{\theta_{i,t}+1}\left(1+\tau_{i,t}\right)e^{-\theta_{i,t}\tau_{i,t}} & & \end{array}$$ (9)

where $\theta_{i,t}$ is the Lindley parameter in segment *i* and quarter *t*.

Eqs (6)–(9) are the conventional expression forms of NB-L model. Lindley distribution can be regarded as a mixture of Gamma distribution and Bernoulli distribution. To facilitate modeling, the NB-L model should be further formulated as a hierarchical model of each distribution.

According to Rusli et al. [15] and Saengthong et al. [20], combined with Gamma distribution and Bernoulli distribution, the hierarchical expression of Lindley distribution is as follows:


$$\begin{array}{ccc} \tau_{i,t}∼\text{Gamma}\left(1+\chi_{i,t},\theta_{i,t}\right),\;\chi_{i,t}∼\text{Bernoulli}(\frac{1}{1+\theta_{i,t}}) & & \end{array}$$ (10)

In summary, the complete hierarchical structure expression of NB-L model is summarized as follows:


$$\begin{array}{cccc} & p\left(Y_{i,t}=y_{i,t},\tau_{i,t},\lambda_{i,t}\right)∼NB\left(y_{i,t},\tau_{i,t}\lambda_{i,t}\right) & & \\ & \lambda_{i,t}=\exp\left(\beta X_{i,t}+\varepsilon_{i,t}\right) & & \\ & \tau_{i,t}∼\mathrm{Gamma}\left(1+\chi_{i,t},\theta_{i,t}\right) & & \\ & \chi_{i,t}∼\mathrm{Bernoulli}\left(\frac{1}{1+\theta_{i,t}}\right) & \end{array}$$ (11)

The core mixture distribution of the NB-GE model can be expressed as a hierarchy of NB and GE distributions. The probability distribution equation for the NB model is:


$$\begin{array}{ccc} p\left(Y_{i,t}=y_{i,t}\right)={\left(\frac{1∕\alpha }{1∕\alpha +{∋}_{i,t}}\right)}^{1∕\alpha }\frac{\Gamma \left(1∕\alpha +y_{i,t}\right)}{\Gamma (1∕\alpha )y_{i,t}!}{\left(\frac{{∋}_{i,t}}{1∕\alpha +{∋}_{i,t}}\right)}^{y_{i,t}} & & \end{array}$$ (12)

where, ${∋}_{i,t}$ is the mixed distribution parameter of segment *i* in time *t*, which can decompose the product of $\lambda_{i,t}$ and $\Lambda_{i,t}$, while $\Lambda_{i,t}∼GeneralizedExponential(\omega ,v)$.


$$\begin{array}{ccc} & {∋}_{i,t}=\lambda_{i,t}\Lambda_{i,t} & \end{array}$$ (13)

$$\begin{array}{cc} & f\left(\Lambda_{i,t};\omega_{i,t},v_{i,t}\right)=\omega_{i,t}v_{i,t}{\left(1-e^{-\omega_{i,t}\Lambda_{i,t}}\right)}^{v_{i,t}-1}e^{-\omega_{i,t}\Lambda_{i,t}} \end{array}$$ (14)

where, $\omega_{i,t}$, $v_{i,t}$ respectively represent the scale parameter and shape parameter of GE distribution in segment *i* within time *t*.

In summary, the complete hierarchical structure expression of NB-GE model is summarized as:


$$\begin{array}{cccc} & p\left(Y_{i,t}=y_{i,t},\Lambda_{i,t},\lambda_{i,t}\right)∼NB\left(y_{i,t},\lambda_{i,t}\Lambda_{i,t}\right) & & \\ & \lambda_{i,t}=\exp\left(\beta X_{i,t}+\varepsilon_{i,t}\right) & & \\ & \Lambda_{i,t}∼\;Generalized\;Exponential\;\left(\omega_{i,t},v_{i,t}\right) & \end{array}$$ (15)

### Innovation crash models considering heterogeneity

The regression coefficients *β*, over discrete parameter *α* and intercept terms *£* of the NB-L and NB-GE models are fixed in all segments and quarters, which cannot fully capture the influence of unobserved factors on the crash frequency. Based on the NB-L model, the following discuss how to set the *β*, *α* and *£* as random parameters to reflect the heterogeneity through their random changes among samples. The models with *α* and *£* randomization are called Random Effect Negative Binomial Lindley (*£*-RENB-L and *α*-RENB-L) models, and the model with *β* randomization is called Random Parameter Negative Binomial Lindley (RPNB-L) model.

According to the instructions provided by Rusli et al. [15], we set $j_{i,t}=1∕(1$  +  $\alpha_{i,t})$ and assume that $j_{i,t}$ follows the Beta distribution to achieve the purpose of normalization and randomization of *α*. Therefore, the hierarchical structure of *α*-RENB-L model can be concluded as follows: as:


$$\begin{array}{cccc} & p\left(Y_{i,t}=y_{i,t};\alpha_{i,t},\tau_{i,t},\lambda_{i,t}\right)∼NB\left(y_{i,t},\tau_{i,t}\lambda_{i,t}\right) & & \\ & \lambda_{i,t}=\exp\left(\beta X_{i,t}+£+\varepsilon_{i,t}\right) & & \\ & \tau_{i,t}∼Gamma\left(1+\chi_{i,t},\theta_{i,t}\right) & \\ & \chi_{i,t}∼Bernoulli\left(\frac{1}{1+\theta_{i,t}}\right) & & \\ & \varepsilon_{i,t}∼Gamma\left(1,\alpha_{i,t}\right) & & \\ & 1∕\left(\alpha_{i,t}+1\right)∼Beta(a,b) & & \end{array}$$ (16)

where, $\alpha_{i,t}$ is the over-discretized parameter of NB distribution of segment *i* in quarter *t*; *£* is the intercept term in the link function.

*£*-RENB-L model assumes that the intercept term ${£}_{i,t}$ in the link function of the NB-L model follows a Normal distribution *N* ( *c* , *d* ) . Specifically, the hierarchical structure of *£*-RENB-L model is as follows:


$$\begin{array}{cccc} & p\left(Y_{i,t}=y_{i,t};\alpha_{i,t},\tau_{i,t},\lambda_{i,t}\right)∼NB\left(y_{i,t},\tau_{i,t}\lambda_{i,t}\right) & & \\ & \lambda_{i,t}=\exp\left(\beta X_{i,t}+{£}_{i,t}+\varepsilon_{i,t}\right) & & \\ & \tau_{i,t}∼Gamma\left(1+\chi_{i,t},\theta_{i,t}\right) & \\ & \chi_{i,t}∼Bernoulli\left(\frac{1}{1+\theta_{i,t}}\right) & & \\ & \varepsilon_{i,t}∼Gamma\left(1,\alpha_{i,t}\right) & & \\ & E_{i,t}∼N(c,d) & & \end{array}$$ (17)

The RPNB-L model introduces a new random component $\omega_{i,t}$, and assumes that $\omega_{i,t}$ follows a Normal distribution to randomize the regression coefficients. Specifically, the hierarchy of RPNB-L model is as follows:


$$\begin{array}{cccc} & p\left(Y_{i,t}=y_{i,t};\alpha ,\tau_{i,t},\lambda_{i,t}\right)∼NB\left(y_{i,t},\tau_{i,t}\lambda_{i,t}\right) & & \\ & \lambda_{i,t}=\exp\left(\beta_{i,t}X_{i,t}+£+\varepsilon_{i,t}\right) & & \\ & \beta_{i,t}=\beta +w_{i,t} & & \\ & w_{i,t}∼N(0,\sigma ) & \\ & \tau_{i,t}∼Gamma\left(1+\chi_{i,t},\theta_{i,t}\right) & & \\ & \chi_{i,t}∼Bernoulli\left(\frac{1}{1+\theta_{i,t}}\right) & & \\ & \varepsilon_{i,t}∼Gamma(1,\alpha ) & & \end{array}$$ (18)

In parameter estimation of RPNB-L model, significant variables whose standard deviation of regression coefficient are significantly close to 0 are defined as random variables, that is, there is heterogeneity in the influence of this variable on crashes. On the contrary, it is considered that there is no heterogeneity in the safety effect of this risk factor.

### Parameter estimation method and performance test index

Based on the clues provided in the literature [24], the candidate models were used for Bayesian parameter estimation using WinBUGS software. Referring to Wen et al. [56], This study used the *N* ( 0 . 01 , 10000 )  as the prior distribution of regression coefficients *β* and $\beta_{i,t}$ for all models. Similarly, the Lindley parameter *θ* is assigned *Gamma* ( 0 . 3 , 0 . 5 ) . The scale parameter *ω* and shape parameter *v* of GE distribution are assigned *Gamma* ( 0 . 01 , 0 . 01 ) . The prior distribution of random variable $j_{i,t}$ of *α*-RENB-L model was set as *B* ( 0 . 5 , 0 . 5 ) . The intercept random variable ${£}_{i,t}$ of *£*-RENB-L model is set as *N* ( 0 . 01 , 10000 ) . In addition, a chain is set for 100000 iterations and the first 70000 iterations are discarded. The convergence of MCMC simulation was judged according to Gelman-Rubin statistic in WinBUGS.

Following the clue provided by reference [23,59], Deviation Information Criterion (DIC) is used as an assessment of goodness-of-fit, while Mean Absolute Deviation (MAD) and Root Mean Square Error (RMSE) together are used as an assessment of prediction accuracy. Specifically, DIC assesses the goodness-of-fit by combining the complexity penalty term and the bias. The MAD denotes the absolute value of the difference between the actual observations and the posterior mean, while the RMSE de-notes the square root of the difference between the actual observations and the posterior mean. The specific calculation method of each performance test index is as follows:


$$\begin{array}{ccc} & DIC=\bar{D}+PD & \end{array}$$ (19)

$$\begin{array}{cc} & MAD=\frac{1}{IXT}\overset{I}{\underset{i=1}{\sum }}\overset{T}{\underset{t=1}{\sum }}|\bar{y_{i,t}}-y_{i,t}| \end{array}$$ (20)

$$\begin{array}{cc} & RMSE=\sqrt{\overset{I}{\underset{i=1}{\sum }}\overset{T}{\underset{t=1}{\sum }}{\left(\bar{y_{i,t}}-y_{i,t}\right)}^{2}∕N} \end{array}$$ (21)

where $\bar{D}$ represents the a posteriori mean deviation of the parameters, which is used to measure the fitting accuracy of the model, and *Pd* is the effective parameter, which is used to measure the complexity of the model. *N*, *I*, *T*, and *C* represent the number of samples, the number of road segments, the number of quarters, and the number of parameters included in the model, respectively. $\bar{y_{i,t}}$ denotes the predicted crash frequency for segment *i* in quarter *t*. $y_{i,t}$ denotes the actual crash frequency for segment *i* in quarter *t*.

In addition, Elastic Coefficients and Marginal Effects were further introduced to quantify the safety effects of significant factors [7]. Specifically, the Elasticity Coefficient was used to describe the percentage change in crash frequency for each 1% increase in the continuous significant variables. The Marginal Effect represented the change degree of the average crash frequency per kilometer for each unit increase of the discrete significant variables. The calculation methods of safety effect quantitative indexes are as follows:


$$\begin{array}{ccc} & E_{x}=\frac{1}{NxT}\overset{N}{\underset{i=1}{\sum }}\overset{T}{\underset{t=1}{\sum }}\left(\frac{àu_{i,t}x_{i,t}}{u_{i,t}u_{i,t}}\right)=\frac{1}{NxT}\overset{N}{\underset{i=1}{\sum }}\overset{T}{\underset{t=1}{\sum }}\left(\beta x_{i,t}\right) & \end{array}$$ (22)

$$\begin{array}{cc} & M_{x}=\frac{1}{NxTxL}\overset{N}{\underset{i=1}{\sum }}\overset{T}{\underset{t=1}{\sum }}\frac{àu_{i,t}}{àx_{i,t}}=\frac{1}{NxTxL}\overset{N}{\underset{i=1}{\sum }}\overset{T}{\underset{t=1}{\sum }}\left[\beta \exp\left(\beta x_{i,t}\right)\right] \end{array}$$ (23)

where, $E_{x}$ is the Elasticity Coefficient of continuous variable x on crash frequency, $x_{i,t}$ is the value of *x* in segment *i* and time period t, *β* is the regression coefficient of $x_{i,t}$, $M_{x}$ is the Marginal Effect of discrete variable *y* on crash frequency, and *L* is the average length of modeling sample.

## Results and analysis

### Super parameters and performance test indicators

**Fig 4:**
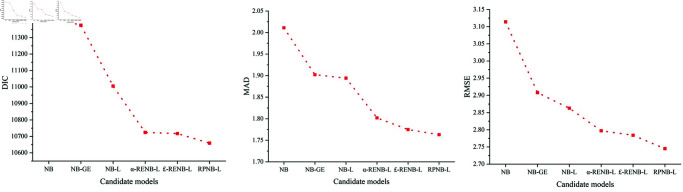
Comparison of the goodness-of-fit of candidate models.

The test results of all candidate models are shown in Table 5 and Fig 4.

(1) Firstly, the effectiveness of the improved models based on excess zeros was considered. The DIC, MAD and RMSE indexes of the NB-L model were 11005, 1.894 and 2.863, respectively, which were better than those of the NB-GE and NB models. The DIC, MAD and RMSE indexes of the NB-GE model were 11375, 1.902 and 2.908, respectively, which were superior to those of the NB model. These results corroborate the findings of Rusli et al. [15] and Cai et al. [7], highlighting the significant advantages of the Lindley and GE distributions in fitting multi-zero-valued crash samples.
(2) When heterogeneity is taken into account, the RPNB-L model achieved the best performance among all candidate models, with the lowest DIC, MAD, and RMSE values. The goodness-of-fit indices for *α*-RENB-L and *£*-RENB-L models have little difference, which were all lower than that of RPNB-L model but better than that of NB-L, NB-GE and NB models. NB model had the worst goodness of fit because it neither considers excess zeros nor heterogeneity. Such results are consistent with Khanal et al. [60] and Hou et al. [33], that the randomization of coefficients was a better way to deal with heterogeneity than the randomization of *α* and *£*.

### Estimation results of regression coefficients

The estimated results for each candidate model are presented in Table 6. As shown in the Table 6, the NB, NB-GE, NB-L, *α*-RENB-L, *£*-RENB-L, and RPNB-L models identified 16, 18, 17, 17, 18, and 18 risk factors, respectively, which were significant at the 95% Bayesian Credible Interval (BCI). All of the significant factors had consistent safety effects across the candidate models, as evidenced by the sign of their regression coefficients. Moreover, the risk factors that were significantly positively correlated with crash frequency included ln(SL), ST, CL, PT, DS, CUR„ SLO, SLL, ln(QADT), IIVE, VVE, and SMR. On the other hand, the risk factors that were significantly negatively correlated with crash frequency included CUL, IVVE, PCI, RQI, SRI, TR, WD, and WP.

### Estimation results of random parameters

**Fig 5:**
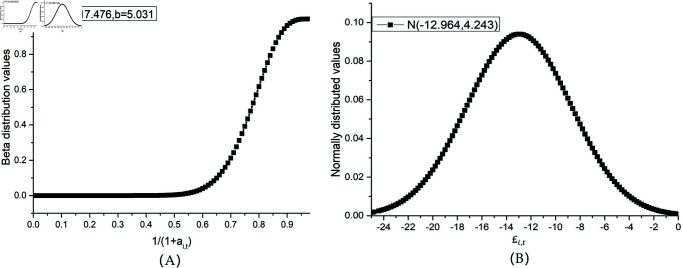
The distributions of random parameters $j_{i,t}$ and ${£}_{i,t}$. (A) Parameter random distribution in the *α*-RENB-L model. (B) Parameter random distribution in the *£*-RENB-L model.

The distributions of random parameters $j_{i,t}$ and ${£}_{i,t}$ are shown in Fig 5. $j_{i,t}$ follows *B* ( 17 . 476 , 5 . 031 ) , and ${£}_{i,t}$ follows *N* ( − 12 . 964 , 4 . 243 ) , indicating that *α*-RENB-L and *£*-RENB-L models can effectively describe partial heterogeneity.

**Fig 6:**
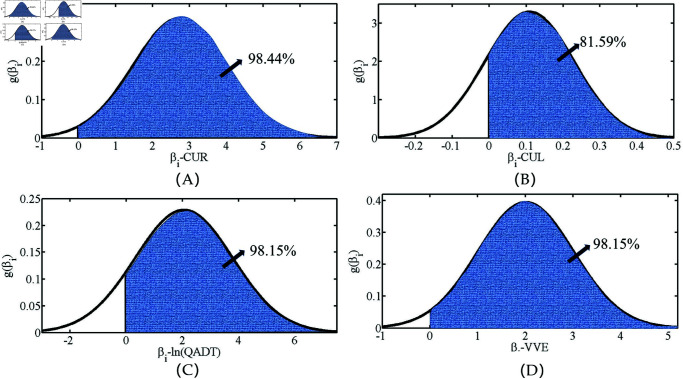
Distribution status of random coefficients in the RPNB-L model. (A) The distribution of regression coefficients in CUR. (B) The distribution of regression coefficients in CUL. (C) The distribution of regression coefficients in ln(QADT). (D) The distribution of regression coefficients in VVE.

The estimation results of the RPNB-L model indicated that the regression coefficients of CUR, CUL, In(QADT), and VVE are all random parameters, as presented in Table 4 and Fig 6. The coefficients of these four variables vary randomly between samples, reflecting unobserved heterogeneity. For example, the regression coefficient for the random variable CUR was detected as following a normal distribution with a mean of 2.746 and a standard deviation of 1.274 (shown in Fig 5a), indicating that CUR was significantly positively correlated with crash frequency in 98.44 % of the samples, while it was significantly negatively correlated with crash frequency in the remaining 1.56 % of the samples. According to Ahmed et al. [52], CUR and CUL interact with unobserved fatigue driving related factors, while ln(QADT) and VVE interacts with weather conditions related factors and design speed.

**Table 4:** Statistical characteristics of random variables.

Random variables	Mean	Std. Err.	95% BCI of Std. Err.
CUR	2.746	1.274	(1.152,4.016)
CUL	0.109	0.121	(0.049,0.137)
In(QADT)	2.054	1.743	(1.104,2.331)
VVE	2.008	1.011	(0.712,1.329)

95% BCI represents the 95% Bayesian credibility interval.

### Analysis of traffic safety effects

**Fig 7:**
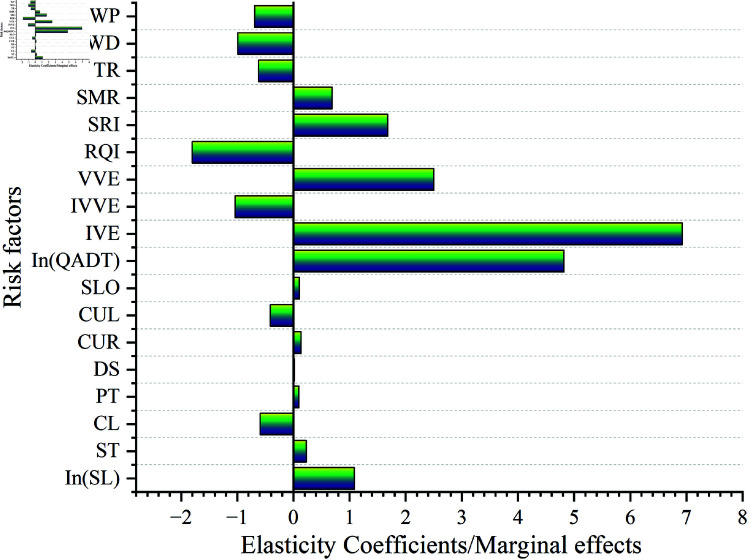
Histogram of elasticity coefficients and marginal effects of risk factors for the RPNB-L model.

Combined with the Elastic Coefficients and Marginal Effect of RPNB-L model (as shown in Table 7 and Fig 7.), the safety effects of factors related to mountain freeway in China were deeply analyzed.

**Table 5:** Hyperparameters and performance indexes of candidate models.

Models	NB		NB-GE		NB-L		*α*-RENB-L		*£*-RENB-L		RPNB-L	
	Mean	95%BCI	Mean	95%BCI	Mean	95%BCI	Mean	95%BCI	Mean	95%BCI	Mean	95%BCI
*£*	-12.475	(-15.274,-9.809)	-12.475	(-15.274,-9.809)	-10.274	(-13.076,-6.988)	-14.942	(-19.477,-10.255)	-12.964	(-16.875,-8.622)	-11.522	(-14.623,-8.905)
*α*	2.579	(2.122,3.014)	3.123	(2.743,3.522)	3.862	(2.518,5.668)	4.194	(3.012,5.571)	4.621	(2.874,5.977)	5.011	(3.723,7.847)
*a*			–	–	–	–	17.476	(13.097,21.866)	–	–	–	–
*b*			–	–	–	–	5.031	3.297,.012)	–	–	–	–
*θ*			–	–	2.576	(1.135,4.517)	4.332	(2.761,5.819)	6.379	(5.091,7.823)	6.993	(5.021,8.774)
*ω*			1.987	(1.063,3.116)	–	–	–	–	–	–	–	–
*v*			1.022	(0.559,1.642)	–	–	–	–	–	–	–	–
DIC	11427		11375		11005		10724		10717		10659	
MAD	2.011		1.902		1.894		1.802		1.775		1.763	
RMSE	3.114		2.908		2.863		2.797		2.784		2.745	

95% BCI represents the 95% Bayesian credibility interval.

**Table 6:** Coefficient estimation results of candidate models.

Models	NB		NB-GE		NB-L		*α*-RENB-L		*£*-RENB-L		RPNB-L	
	Mean	95%BCI	Mean	95%BCI	Mean	95%BCI	Mean	95%BCI	Mean	95%BCI	Mean	95%BCI
In (SL)	2.149	(2.108,2.193)	1.071	(0.456,1.522)	1.444	(1.153,1.758)	1.480	(1.078,1.868)	1.374	(1.175,1.594)	1.484	(1.102,1.872)
ST	0.025	(0.019,0.031)	0.114	(0.079,0.143)	0.050	(0.031,0.089)	0.079	(0.034,0.106)	0.123	(0.096,1.411)	0.115	(0.077,0.139)
CL	-0.658	(-0.816,-0.493)	-0.487	(-0.717,-0.123)	-0.442	(-0.746-,0.119,)	-0.652	(-1.24,-0.007)	-0.448	(-0.726,-0.159,)	-0.73	(-1.323,-0.145)
PT	0.128	(0.105,0.153)	0.074	(0.021,0.108)	0.064	(0.029,0.094)	0.071	(0.032,0.115)	0.072	(0.048,0.098)	0.085	(0.042,0.120)
DS	0.110	(0.085,0.133)	0.322	(0.176,0.451)			0.102	(0.031,0.189)	0.028	(0.004,0.051)	0.111	(0.037,0.188)
CUR	1.174	(0.996,1.365)	2.243	(1.632,2.977)	1.456	(1.077,1.787)	2.272	(1.596,2.907)	1.877	(1.455,2.332)	2.746	(1.712,3.678)
CUL	-0.130	(-0.141,-0.119)	-0.375	(-0.412,-0.309)	-0.456	(-0.607,-0.316,)			-0.441	(-0.563,-0.314)	-0.109	(-0.172,-0.046,)
SLO	0.019	(0.007,0.032)	0.041	(0.029,0.051)	0.061	(0.032,0.087)			0.099	(0.077,0.126)	0.107	(0.014,0.147)
SLL			1.077	(0.821,1.265)	1.179	(0.742,1.561)						
In(QADT)	3.860	(3.794,3.921)	2.991	(2.073,3.765)	3.299	(2.400,3.977)	1.568	(1.278,1.915)	3.522	(3.239,3.798)	2.054	(1.627,2.559)
IVE							-2.638	(-4.977,-0.106)			-2.184	(-4.858,-0.210)
IIVE	9.307	(9.192,9.432)					9.611	(4.522,16.041)	18.56	(9.497,28.420)		
IVVE	-44.038	(-50.670,-39,782)	-48.33	(-55.41,-39.87)	-44.31	(-51.87,-35.53)	-61.82	(-86.00,-38.44)	-41.48	(-46.750,-35.820)	-64.58	(-86.88,-41.39)
VVE	1.635	(1.516,1.747)	3.922	(2.976,4.729)	5.581	(4.095,7.345)	2.053	(1.040,2.998)	6.53	(1.952,9.878)	2.008	(1.150,2.910)
PCI			-0.009	(-0.012,-0.006)	-0.016	(-0.023,-0.006)	-0.006	(-0.012,-0.002)	-0.041	(-0.048,-0.033)		
RQI									-0.048	(-0.066,-0.034)	-0.004	(-0.029,-0.002)
SRI	-0.912	(-1.273,-0.692)	-0.064	(-0.091,-0.024)	-0.033	(-0.069,-0.004)	-0.061	(-0.118,-0.002)			-0.054	(-0.117,-0.001)
SMR	3.179	(2.691,3.712)	2.012	(1.677,2.509)	1.616	(1.240,2.041)	2.053	(1.040,2.998)	1.424	(0.930,1.905)	2.008	(1.150,2.910)
TR	-5.271	(-6.442,-4.116)	-6.175	(-7.812,-4.916)	-5.864	(-7.278,-4.286)	-4.868	(-9.007,-1.040)	-6.571	(-8.045,-5.060)	-5.051	(-8.441,-1.519)
WD			-2.943	(-2.033,-1.476)	-1.177	(-1.718,-0.495)	-2.561	(-3.162,-2.302)	1.558	(0.273,2.062)	-2.363	(-2.740,-2.057)
WP	-2.074	(-2.732,-1.510)	-1.791	(-2.654,-1.110)	-0.808	(-1.497-0.070)	-1.500	(-1.794,-0.527)	-2.767	(-4.147,-1.378)	-1.555	(-2.043,-0.775)
Intercept	-1.974	(-2.223,-1.597)	-1.375	(-1.742,-0.431)	-1.106	(-1.681,-0.327)	-1.449	(-3.368,-0.012)			-1.799	(-2.431,-0.549)

95% BCI represents the 95% Bayesian credibility interval.

#### Traffic safety effects of design features.

The elasticity coefficient of ln(SL) was 1.09, indicating an approximately linear positive correlation with crash frequency. The artificial segment length had no meaningful safety effects; that is, longer segments lead to more natural crashes without the influence of other factors [5,61]. According to the Marginal Effect of ST, the crash frequency of tunnel segments, service areas, and interchange segments was, on average, 0.23 crashes/km higher than that of basic segments. The presence of abundant tunnel segments on China’s mountainous freeways, characterized by frequent sudden environmental changes (e.g., rapid changes in vision, lighting, etc.), increases the probability of nervous driving and sharp reactions to light and dark transitions, thereby raising crash risks [46]. Additionally, the presence of frequent diversion and merging behaviors in the service areas and interchange segments with high traffic volumes inevitably increases the interaction behavior of vehicles thereby increasing the risk of crashes [1,7]. The Marginal Effect of CL showed that the provision of climbing lanes reduced crash frequency by an average of 0.59 crashes/km, as climbing lanes separate slower heavy vehicles from the general traffic flow, thereby increasing the stability of arterial traffic [3]. The Marginal Effect of PT revealed that crash frequency in cement concrete segments was, on average, 0.1 crashes/km higher than in asphalt concrete segments. This suggests that the lower noise levels and greater smoothness of asphalt pavement contribute to improved safety. It is also important to note that the basic segments in this study are asphalt-paved, while tunnel and service segments are paved with cement concrete, which exacerbates the negative safety effects of cement concrete pavements [5,6]. The Marginal Effect of DS indicated that segments with a design speed of 120 km/h had an average crash probability of 0.02 crashes/km higher than segments with a design speed of 100 km/h. In China’s mountainous freeways, higher design speeds lead to more complex vehicle interactions, thus increasing crash probability [56,62]. Among the flat and longitudinal indicators, each 1% increase in the CUR and SLO indicators will increase the crash frequency by 0.14% and 0.11% respectively. A 1% increase in the CUL indicator would reduce the crash frequency by 0.41%. The sharp curved segments resulted in severely restricted sight distances and compressed the turning reflection time of high-speed vehicles, greatly reducing the tolerance of driving safety and thus increasing the risk of crashes [63]. A longer curve length reduces curvature, giving the driver more time to react and maneuver, thereby enhancing safety levels [22]. Steep segments demand high braking performance to ensure vehicle safety. Additionally, in traffic systems with a high volume of heavy vehicles, the speed differential between heavy and standard vehicles exacerbates traffic flow instability in longitudinal slope segments, which is prone to crashes [64].

**Table 7:** Elasticity coefficients and marginal effects of RPNB-L model.

variables	Means	Std. Err.	95%BCI	
In (SL)	1.09	0.15	1.80	2.39
ST	0.23	0.02	0.16	0.29
CL	-0.59	0.17	-0.92	-0.26
PT	0.10	0.02	0.07	0.14
DS	0.02	0.01	0.00	0.04
CUR	0.14	0.03	0.08	0.21
CUL	-0.41	0.07	-0.55	-0.28
SLO	0.11	0.01	0.08	0.13
In (QADT)	4.82	0.33	4.18	5.47
IVE	6.93	0.87	5.24	8.63
IVVE	-1.04	0.13	-1.29	-0.80
VVE	2.50	0.22	2.07	2.94
RQI	-1.80	0.94	-3.65	0.04
SRI	1.68	0.52	0.66	2.70
SMR	0.69	0.11	0.48	0.90
TR	-0.62	0.05	-0.73	-0.52
WD	-0.99	0.12	-1.31	-0.88
WP	-0.69	0.15	-1.04	-0.24

95% BCI represents the 95% Bayesian credibility interval.

#### Traffic safety effects of traffic conditions.

The results indicated that an increase in traffic volume leads to a higher crash risk. Specifically, a 1% increase in ln(QADT) results in a 4.82% increase in crash frequency. This result was verified to be applicable to freeways, urban trunk roads and rural trunk roads [8,13,42]. Strong interactions within traffic systems with high traffic flow contribute to negative safety effects. Regarding traffic composition, increases in IVE and VVE were significantly associated with higher crash frequency, whereas IVVE showed the opposite effect. Specifically, a 1% increase in IVE and VVE results in a 6.93% and 2.50% increase in crash frequency, respectively, while a 1% increase in IIVE leads to a 1.04% decrease in crash frequency. Potential reasons [7] for such results were that (1) The number of Class I vehicles in this study accounts for the majority (74.1%) of the total number of vehicles, whose influences on crashes were dominant. Segments with more class 1 vehicles are bound to have more interaction effects, frequent overtaking and lane change behaviors, thus increasing the possibility of crashes. (2) The slow speeds, large volumes and slow braking responses of heavy vehicles lead to the large speed difference between vehicles, limited vision of standard cars and other unstable factors in the traffic system, which have safety risks. (3) The negative correlation between Class IV vehicles and the crash frequency may be attributed to the drivers’ route familiarity and driving skills.

#### Traffic safety effects of pavement performance.

Both RQI and SRI were significantly and negatively correlated with crash frequency. Specifically, each 1% increase in RQI and SRI was associated with a 1.8% and 1.68% decrease in crash frequency, respectively. These findings align with those of Ahmed et al. [52] and Alnawmasi et al. [58] and reflect real-world conditions, where good driving comfort and skid resistance typically contribute to enhanced driving safety. This highlights the critical importance of routine road maintenance for improving traffic safety.

#### Analysis of traffic safety effects of weather conditions.

The impact of precipitation levels on crashes varied. SMR showed a significant positive correlation with crash frequency, while TR demonstrated a significant negative correlation. Specifically, a 1% increase in the SMR and TR indicators resulted in a 0.69% increase and a 0.62% decrease in crash frequency, respectively. The reasons for these results include: (1) Rainy conditions reduced drivers’ visibility and limited the space available for safe operations; (2) Rainy weather decreased the adhesion between the road surface and tires, particularly in segments with higher volumes of heavy vehicles, increasing the likelihood of loss of control for these vehicles. One possible explanation for the negative safety effects of TR is that heavy rain disrupts travel plans, leading to lower traffic volumes on freeways during heavy rain, which in turn reduces the probability of crashes [53]. Additionally, both WD and WP are negatively correlated with crash frequency. Specifically, a 1% increase in the WD and WP indices would result in a 0.99% and 0.69% decrease in crash frequency, respectively. Although there are no studies on the safety effects of these factors, these findings are consistent with the conditions of China’s mountainous freeways. Regarding driver behavior, some drivers avoid driving in complex mountainous freeway environments during sustained and strong wind conditions, which leads to reduced traffic volumes and, ultimately, a decrease in crash frequency.

## Conclusions

This paper gradually adopted the NB-GE and NB-L models, considering excess zeros, and also the *α*-RENB-L, *£*-RENB-L and RPNB-L models, considering both excess zeros and heterogeneity, to analyze the causes of crashes on mountainous freeways in China, including freeway design features, traffic volume and composition, pavement performances, and weather conditions. This study is the first to apply innovative crash frequency models, considering both excess zeros and heterogeneity, to analyze the safety of mountain freeways in China. It represents the first comprehensive safety evaluation of Chinese mountain freeways based on multidimensional information of human-vehicular-road-environment.

The findings of this study offer novel insights for policy-making by freeway administrators. Specifically: (1) The risk implications of large vehicles in safety management require urgent attention. We strongly recommend conducting a comprehensive benefit-cost analysis of safety improvement measures targeting large vehicle control on mountainous highways in China. Should the benefit-cost ratio prove favorable, practical intervention strategies for large vehicle regulation should be prioritized to effectively mitigate economic losses and reduce traffic-related casualties. (2) Concerning the safety effects of pavement performance, maintenance measures targeting enhanced surface evenness, improved skid resistance, or reduced pavement distress demonstrate quantifiable safety benefits. A critical implication of this discovery lies in budgetary optimization: under fiscal constraints, highway authorities should strategically prioritize these pivotal performance indicators while selectively allocating residual funds to secondary pavement characteristics based on cost-effectiveness analyses. (3) The integration of high-resolution meteorological data significantly enhances the predictability of safety management systems, enabling transportation agencies to deploy adaptive control strategies. For instance, proactive traffic control protocols could be activated in critical zones (e.g., tunnels, intersections, and service areas) prior to light-to-moderate rainfall events. Notably, this study revealed a counterintuitive safety improvement during heavy precipitation-a phenomenon potentially attributable to reduced travel demand and heightened driver vigilance under extreme weather conditions. Consequently, resource-constrained authorities should prioritize preventive measures during light/moderate rainfall phases while maintaining robust emergency response capabilities for heavy precipitation scenarios.

The results of this study contribute to a better understanding of crash causation on China’s mountain freeways and to the development of reliable safety countermeasures for the world’s largest mountain highway system. The following topics warrant further study:

(1) To improve the reliability of safety analysis, modeling methods based on internal attributes such as temporal-spatial correlation and endogeneity of crash data structures need to be studied.
(2) The safety effects of overpasses, tunnels, and service areas need to be further refined. This includes analyzing traffic volume of on-ramp and off-ramp, acceleration and deceleration lane length, interchange length, ramp speed limit, tunnel length, tunnel spacing, and other relevant factors.
